# The Institutional Worker

**Published:** 1913-02-22

**Authors:** 


### INSTITUTIONAL WORKER

**Being a Special Supplement to "The Hospital"**

### Nervous Breakdown.

You cannot do better than apply to A. C. Holland, Esq., Hon. Secretary of the St. Moritz Aid Fund, 8 The Orchard, Bedford Park, London, W. The fees charged are five francs a day, and include medical attendance and every comfort. The Home is intended for gentlefolk of small means who are earning their own living and who are threatened with lung or nerve trouble from overwork or other causes. You will have to be passed as suitable by one of the London doctors, and also to pay your own travelling expenses to St. Moritz.—Beta.

### Hospital Almoners' Training.

Full information can be obtained from the Hospital Almoners' Council, Denison House, Vauxhall Bridge Road, London, S.W.—C. C.

### Overworked Priest.

The Clergy Seaside Rest, 4 and 5 Royal Crescent, Margate, would perhaps prove suitable. Application should be made to the Rev. Michael Pryor, D.D., Holy Trinity Vicarage, Margate. The terms are 25s. a week.—Parishioner.

### Low Terms for Children's Homes.

If you intend to cover the entire expenses of the Home out of patients' payments, it is hardly possible you could succeed at a lower charge than 15s. a week. If, however, you are already housed and merely intend to reckon the domestic expenses, then we agree with you that from 8s. to 10s. a week might suffice. Do not embark on any scheme foredoomed to failure from forming an erroneous estimate of running expenses.—B. T.

### Nursing in Buenos Aires.

The demand for British nurses is limited by the fact that a knowledge of Spanish is an essential qualification for nurses, and few Englishwomen will take the trouble to master any foreign language colloquially. The British Hospital, Calle Perdial, Buenos Aires, trains its own nurses. Other requirements are met by Mrs. Waddington's Maternity and General Nursing, Cochabamba, and by Mrs. Murray's Nursing Co-operation, 1635 Callao, Buenos Aires. Apply to the latter for details about employment; but we warn you that without Spanish little can be done.—Prudentia.

### OUR BUREAU OF INFORMATION.

### Rules for Correspondents.

1. Every letter, on whatever subject, must be accompanied by the coupon to be cut from the back cover (inside page) of The Hospital, current issue, and must contain the name and address of the correspondent with pseudonym for publication if desired.
2. Replies by post cannot be given, save under exceptional circumstances at the Editor's discretion.
3. Letters from Approved Homes in reply to special needs published in the Bureau should state terms and full particulars, and be sent prepaid under cover to the Editor of the Bureau with name written across coupon for identification.
4. Proprietors of Homes which have not yet been entered on the List of Approved Homes, but have spare accommodation likely to suit special needs, are invited to write for an application form for registration. The fee for registration, which includes two announcements of the Home in the Bureau and other privileges, is 5s.
5. All communications to be addressed to the Editor of The Hospital, 28 Southampton Street, Strand, London, W.C., and marked "Bureau of Information."

### Invitation to Readers.

**The Editor is prepared to forward to applicants, without fee on either side, offers of accommodation from Homes on the Approved List suitable for medical and surgical cases, for the aged, for chronic or mental patients, for electrical treatment and massage, for convalescents and for children, at varying terms, in any required locality. Reduced terms can often be arranged. The special needs of any who may be sick or in difficulties are also made known in these columns free of charge.**

### Approved Homes Recently Added to List.

Note.—*No Home is added to the List without medical and other testimony to its good management.*

**Berkshire.**—Rose Cottage, Cholsey, Wallingford. Principal: Sister Marion. Medical and slight mental cases. Aged people; delicate children. Comfortable Home in large garden at the foot of the Berkshire Downs.

**Newbury.**—The Cottage, East Ilsley. Principal: Miss Goodley. Medical, surgical, slight mental, rest-cure cases, massage. Specially adapted for nervous, convalescent, and outdoor treatment. Nice garden.

**Sussex.**—"Melrose," 77 St. Aubyns, Hove. Principal: Miss Thomas. Medical, maternity, rest-cure, and convalescent surgical cases. Private rooms for each patient. Large garden.

**Wimbledon.**—The Alexandra Homes, 153 Merton Road and 4 Francis Grove, St. George's Road. Principal: Mrs. Perrin, with Miss Harriet Perrin. For chronic, senile, and bed-ridden patients in indigent circumstances, who can be received at from 10s. 6d. a week. Doctor attends Home. Garden.

### The Care of Rheumatic Children.

The difficulty about securing this class of patient is that persons who can afford to pay at a remunerative rate prefer to nurse their sick child at home. The school doctors, nurses, and health

### Temporary Home Wanted.

Your district case is a very sad one. We understand that the man, aged sixty-five, is afflicted with rheumatoid arthritis and epithelioma of face, and that his wife, who ordinarily attends on him with your daily help, is compelled to leave him in order to undergo an operation for cataract at Westminster Hospital. His wound makes him ineligible for ordinary chronic Homes, but he is able to get up with a little help. As he can afford to pay 10s. a week, we trust one of our kind friends may offer to receive him during this anxious period. We note he is highly respectable. Letters forwarded to Nurse R. C. (152x).

### Lectures to Nurses.

You will find "The Nurses' Materia Medica," by Herbert French, M.D. (The Scientific Press, 28-29 Southampton Street, Strand, London, 2s. 9d.), exactly what you require for this purpose. Break your matter up into small sections and make the lessons less lectures than instructions, with every fact clearly stated on the blackboard for your pupils to copy into their notebooks and learn by heart.—W. L. Y., Ph.C.

### On Making Omelets.

You say you have tried many recipes and can never get a good omelet made in your house. Few English cooks seem to distinguish themselves with an omelet, and the only way to teach them is to practice making a good omelet yourself until you have found out exactly where and how mistakes are made and the true secret of success. The following are the chief methods adopted by the inexperienced cook to turn her omelets to leather: First, she is apt to mix flour or some other thickening with the eggs, and thus produce a pancake or heavy slab. Secondly, she leaves off beating the eggs some little time before she pours them into the pan, with the result that the air evaporates and the liquid has no rising power. Thirdly, she pours the eggs into lukewarm instead of very hot butter. Fourthly, she fails to seize the exact moment when the omelet is cooked, and either burns it—a flagrant fault—or sends it up wet and sticky. Fifthly, she is heavy handed with the salt, and the delicate flavouring, which the French produce with a "bouquet," is altogether omitted.—Dora.

### To Clean Marble.

You will find the following mixture very satisfactory for this purpose. Ordinary soap is no good. Pound 2 oz. of washing soda, 1 oz. of precipitated whiting, and 1 oz. of pumice stone powder in a mortar. Next mix with them enough boiling water to make the mixture like good cream. Keep in a tightly-corked bottle. Put it on with a small stiff brush, leave it on until dry, and then wash it off with hot water. Dry well and polish with a clean soft cloth.—SISTER K.

### Well Served Meals.

WE can thoroughly sympathise with your difficulties in getting the table well laid and quickly cleared in an establishment where few maids can be afforded and where the kitchen premises are cramped. The long bare room available for meals does not sound attractive. We suggest that you have one side fitted up with a shallow dresser such as any carpenter could make you, at small cost, to one of the old simple designs. Have it stained dark brown, and keep all the china, plates, and dishes ready to use on it. It will brighten the room and save much labour. In cupboards beneath store the plate and knives used at table, with the salt-cellars, table napkins, table cloths, and other accessories. Ask your nurses to take charge, six at a time, of the table. One would have the linen; another the spoons, forks, and knives; a third the plates; a fourth the glasses and drinking water; a fifth the salt, pepper, etc., the bread, butter dishes, and preserves; a sixth the decoration, which may be with ferns, wild flowers, sprays of green, or whatever the garden or woods can furnish. Under this plan the six nurses during their week of service assemble a quarter of an hour before the hour for the meal and set to work. With a little practice it is found that all the necessary details can be deftly got through in less than this time, and great interest is experienced by those who undertake to make the table look nice for the rest. After the meal is over the process of tidying can be rapidly carried out by the same hands; the table is cleared as if by magic, while whatever needs washing is placed on a dinner wagon to be removed by the maids and subsequently replaced. A place for everything is essential to success, and great nicety must be aimed at, for the charm of doing things oneself is to do them better.—"WORRIED."

### The Care of Rheumatic Children

*(continued).*

visitors, who discover these cases among elementary school children, are faced with complete lack of funds wherewith to treat them. One of the factors which retard the extension of school clinics is the underlying conviction that these clinics to be successful must be able to fall back upon Homes where children suffering from incipient consumption, heart weakness, and rheumatism can have prolonged treatment. We are taking steps to bring the Homes which offer such accommodation to the notice of the authorities.—M. P. and CHRONOMETER.

### Schools for Tuberculous Children.

There are no "open-air schools for tuberculous children in the home counties within easy reach of London, in which education is carried on, where all the children are under medical supervision and where fees either cover actual cost or are nominal." There is a sanatorium for children at Holt, Norfolk, where early cases are received at 7s. 6d. a week. Address the Secretary, 68 Denison House, Vauxhall Bridge Road, S.W. You might also write to the Matron, Children's Home, Tatsfield, Surrey, where cases are received at low terms.—E. D. C.

### The Editor's Letter-Box.

**Communications have been received and attended to from:**—Mr. E. G., Littlehampton; Mr. E. P., Hollington; Miss M. E. G., Cholsey; Sister G., Abingdon; Miss A. L., Newmarket; Sister-in-Charge, St. Bridget; Miss Campbell; "Ethel."

The Editor is unable to introduce patients to any but the Homes on the Approved List.—MISS WARE, MISS TILL.

We are much obliged to you for forwarding information for us to the Commercial Intelligence Branch of the Board of Trade, from whom we hope shortly to receive it.—H.M. CHARGÉ D'AFFAIRES, La Paz.

Glad to hear you have had satisfactory answers to your request.—A. L.

### Housekeeping Questions for February.

1. State the quantity of potatoes used in your institution during the month of December, and the price per cwt. paid for them. Enumerate all other kinds of vegetables, cooked or raw, which were served during this month. Was any of this produce grown in the institution gardens? Please state the number of beds.

2. Give the diet for convalescent patients in your institution, and state what it costs per week at the present prices.

The figures must be the result of actual observation and computation, not estimates. Either or both of the questions may be answered by each contributor. The best answers will be published, and for each contribution considered by the Editor to be of sufficient interest for publication payment of FIVE SHILLINGS will be made.

RULES.

The following rules must be observed by all those answering the Housekeeping Questions:—

1. Answers must be written on one side of the paper. They must bear the name and address of the sender and be accompanied by coupon to be cut from the back cover (inside page) of the current issue of THE HOSPITAL. A pseudonym must be chosen if the name is not to be published.

2. Answers must be addressed to the Editor of THE HOSPITAL, 28-29 Southampton Street, Strand, London, W.C.; they must be marked in the left-hand corner "Facts and Figures," and must be received before the end of the month.

### Orange Marmalade.

As you ask for a recipe which gives a solid conserve without too much jelly, try this method: To every twelve Seville oranges allow two lemons. Cut the oranges in quarters, take out the pips, cut all into very fine chips. Allow to every pound of pulp three pints of water. Let it stand for twenty-four hours. Then simmer it gently for half an hour. Let it stand again for twenty-four hours. Then weigh it once more. To every pound of pulp and liquid add 1 lb. of sugar. Put all back into the pan and boil it very fast till it thickens, which will be in an hour or two. Let it get cold before tying it down. A very rich variety can be made with tangerine oranges in the proportion of two Seville to one tangerine.—ELIZA.

### Domestic Science Course.

As you cannot afford a residential training or a collegiate course, we recommend you to apply to the Battersea Polytechnic Department of Domestic Science, where there is a year's course suitable for those who are preparing to be housekeepers. The fees are very low, and the instruction given is quite first-rate.—SPENCER.

### Insight into Matron's Duties.

OPPORTUNITIES are offered both at St. Thomas's and at Guy's Hospital to matrons of Colonial and other institutions, who desire to study the working of large institutions. Write, stating previous training and experience to the Matron at either of these hospitals, and ask for particulars. The instruction given comprises the organisation of the ward staff, the management of the domestic side, kitchen, etc., and the general superintendence of the training school.—EXILE.

### Save the Dregs.

IT is not at all mean, but very right and proper that your economical soul should be exercised about the half-glass of wine left over lest patients should complain of dregs. You should keep a kitchen bottle at hand in which these precious remains can be corked down. They will become clear by standing, and will be of the utmost service to the cook for flavouring jellies, soups, etc.—J. S. P.

